# Crosstalk between Body Microbiota and the Regulation of Immunity

**DOI:** 10.1155/2022/6274265

**Published:** 2022-05-19

**Authors:** Carolina Rojas, Felipe Gálvez-Jirón, Javiera De Solminihac, Cristina Padilla, Ignacio Cárcamo, Natalia Villalón, Mónica Kurte, Karina Pino-Lagos

**Affiliations:** ^1^Facultad de Medicina, Centro de Investigación e Innovación Biomédica, Universidad de los Andes, Santiago, Chile; ^2^Facultad de Odontología, Universidad de Chile, Santiago, Chile; ^3^IMPACT, Center of Interventional Medicine for Precision and Advanced Cellular Therapy, Santiago, Chile

## Abstract

The microbiome corresponds to the genetic component of microorganisms (archaea, bacteria, phages, viruses, fungi, and protozoa) that coexist with an individual. During the last two decades, research on this topic has become massive demonstrating that in both homeostasis and disease, the microbiome plays an important role, and in some cases, a decisive one. To date, microbiota have been identified at different body locations, such as the eyes, lung, gastrointestinal and genitourinary tracts, and skin, and technological advances have permitted the taxonomic characterization of resident species and their metabolites, in addition to the cellular and molecular components of the host that maintain a crosstalk with local microorganisms. Here, we summarize recent studies regarding microbiota residing in different zones of the body and their relationship with the immune system. We emphasize the immune components underlying pathological conditions and how they interact with local (and distant) microbiota.

## 1. Introduction

The study of chronic diseases (CD), now part of modern society, indicates that the individual genetic component plays a minor role in the development of them because only 20 years or so have passed since their appearance. However, the genetic component of the microorganisms that cohabit our body could indeed have evolved during these two decades, suggesting a possible association with the emergence of CD. Many of these multifactorial pathologies associate with an alteration of microbiota's composition and/or diversity, which is known as dysbiosis.

The immune system has been identified as one of the main regulators of normal and dysbiotic microbiota. This has been proved by studying the colonization of bacteria in wild-type animals or in pattern recognition receptor (PRR) deficient mice. PRR deficient animals, either lacking Toll-like receptors (TLRs) or nucleotide-binding oligomerization domain-like receptors (NODs), have shown differences in the composition of intestinal microbiota and in the production of antimicrobial peptides (defensins). Similarly, it has been demonstrated that the production of immunoglobulin-type A (IgA) and the number of B cells are diminished in germ-free mice, suggesting the existence of crosstalk between the host's microbiota and the immune system. In other words, recognition of microorganisms by host cells will determine the inflammatory response or the regulation and maintenance of homeostasis. Likewise, it has been reported that under immunodeficiency, the host also contains a dysbiotic microbiota [1, 2].

In this review, we present definitions of the microbiome and microbiota, and concisely report about microbiota in different zones of our body, and its interaction with the immune system under healthy and pathological conditions.

### 1.1. Microbiome versus Microbiota

In 2019, a discussion about the definition of microbiome took place during a workshop held in Austria, with results reported by Berg and colleagues [3]. This manuscript is highly recommended for those interested in the details of this and other concepts involving microbial ecology. The final definitions are a result of a survey offered to the workshop participants, with the purpose to establish international research standards (one of the goals of the Microbiome Support project).

In the 19^th^ century, it was recognized that an association between the natural environment and microorganisms exists, and for the first time, it is accepted that microorganisms have beneficial effects on the host. The emergence of technological advances allowed the identification and study of microorganisms, highlighting the contribution of the multiomics approaches. In 1988, Whipps and colleagues defined microbiome as “a characteristic microbial community in a reasonable well-defined habitat which has distinct properties and functions and its interactions with microenvironment, resulting in the formation of specific ecological niches” [4]. This definition is the most accepted, and the survey's participants suggested adding amendments to cover the following points: (1) the members of the microbiome, (2) their interactions, (3) spatial and temporal characteristics, (4) the core microbiota, (5) functional prediction and their phenotype, and (6) the interaction between the microbiome and the host.

Clearly, the definition of microbiome does not only refer to the genetic component of the microorganisms but also includes ecological concepts that reflect its complexity. On the other hand, microbiota is understood as the members composing the microbiome, which includes stable microorganisms and those associated with a specific state (also known as intermittent microorganisms). Currently, the microbiota is defined based on DNA sequences with taxonomic information.

In the following sections, we present the most relevant studies linking the microbiota found at specific zones of the body and its interaction with the host's immune system.

### 1.2. Ocular Microbiota

From the first studies on human microbiota, diverse tissues have been investigated, and the eye is not an exception. Initially, it was thought that the ocular microbiota was small because experiments of culturing colonizing agents, those prepared from ocular samples, did not show significant bacterial growth. Currently, thanks to bacterial 16S RNA sequencing, it has been suggested that ocular microbiota is more abundant and varied than was considered [5]. The most relevant bacterial populations found at this location are *Haemophilus*, *Streptococcus*, *Staphylococcus*, *Propionibacterium*, and *Corynebacterium* [5–8]. Other reports have indicated that fungi are also present, highlighting 5 genera: *Malassezia*, *Rhodotorula*, *Davidiella*, *Aspergillus*, and *Alternaria*, which constitute more than 80% of total fungi [9]. Furthermore, some viruses, such as herpes simplex virus and hepatitides B and C, among others, can be considered as well [10].

Presently, it is accepted that ocular microbiota plays a key role in the eye's health, as it is exemplified in the term coined by Zhang et al., as “the ocular surface microenvironment” (OSM), which is used to illustrate the complexity of the ocular network. Among the components of the OSM, systemic hormones, tears, microbiota, and immune cells are included [11]. The OSM functions similarly to the gastrointestinal system and its microbiota, coordinating functions to preserve health, such as inhibition of inflammation, tissue regeneration, and the maintenance of immune tolerance [11]. Immune cells in epithelial tissue cohabit with commensal microbiota in a healthy individual; however, in a pathological state, this situation is broken.

**Figure 1:**
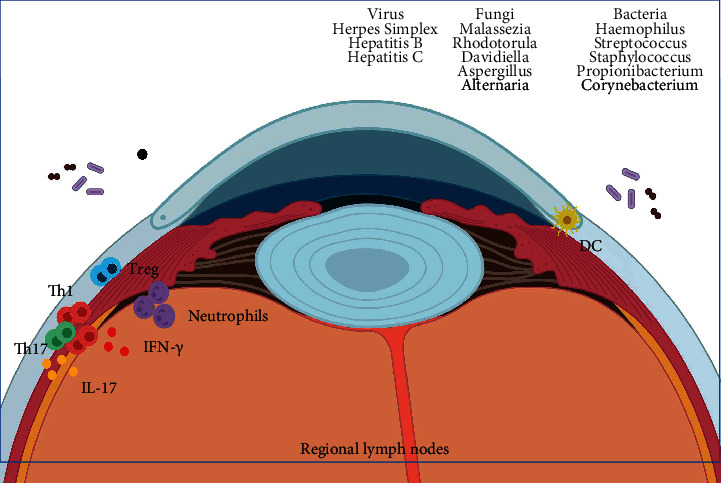
Ocular microbiota. The eye and its components, such as conjunctiva, surrounding skin, and lashes, contained a plethora of microorganisms composing the microbiota of this organ. Several families of virus, fungi, and bacteria are present in distinct areas, and their homeostasis with the host cells is pivotal to keep this zone healthy. Immune components are present as well, with proinflammatory cytokines such as IFN-*γ* and IL-17, and leucocytes like DC, Th1, Th17, Treg, and neutrophils as important factors involved in dysbiosis and eye pathologies.

In diseases such as dry eye, there is an epithelial disruption that activates the innate immune system. A potential explanation for this observation is the presence of TLR5 only at the base of corneal epithelial cells (and not on the surface), whereas if the epithelium remains intact, the microbiota will not interact with TLR5; thus, no activation of the immune system takes place [12]. Another preliminary piece of evidence suggests that treatment with probiotics containing *L. acidophilus* and administered as eye drops could decrease inflammation and symptomatology of vernal keratoconjunctivitis; however, more information is needed to clarify the observed effects [13]. Other example of interaction between ocular microbiota and the immune system in the eye is the study of Leger et al., in which the presence of *Corynebacterium mastitidis* is associated with stimulation of *γδ* T cells that, by producing IL-17, recruit neutrophils to the site. Also, the authors observed that *C. mastitidis* protects the ocular surface from fungi and pathogenic bacterial infections [14]. Another interesting topic is the establishment of a relationship between microbiota (other than ocular) and prevalent ophthalmologic diseases, such as uveitis in which alterations of the intestinal microbiota could influence this disorder [11]. The pathogenic mechanisms driving these diseases are usually involved with a pathogenic response of the immune system [13, 15]. Among these studies, we highlight the report by Chen et al. which describes that the characteristically neurodegenerative damage of glaucoma could be mediated by heat shock proteins- (HSP-) specific T cells. Interestingly, this response does not take place in animals lacking commensal microbiota [15].  Figure 1 summarizes these findings.

### 1.3. Oral Microbiota

The oral cavity contains one of the most diverse microbiotas of our body [16]. It is composed of viruses, protozoa, archaea, fungi, and bacteria, forming extremely complex networks. There are approximately 1,000 described species associated with oral microbiota whose information can be accessed on the Human Oral Microbiome Database (HOMD), extensively described in [17]. In a healthy oral cavity, most habitats are dominated by *Streptococcus*, followed in abundance by *Haemophilus* in the buccal mucosa, *Actinomyces* in the supragingival plaque, and *Prevotella* in the subgingival plaque [16]. In general terms, at least 5 niches can be identified: dental tissue (nonshedding surfaces), saliva, tongue, the gingival crevice/periodontal pocket, and the remaining epithelia of the oral mucosa (shedding surfaces) [18, 19]. Interestingly, nonshedding surfaces harbor microbial communities adhered to the dental surface, building a very ordered and complex form of organization: biofilm [20].

In the oral cavity, under homeostatic conditions, the immune system keeps an effective surveillance without triggering an exacerbated inflammatory response, tolerating commensal microorganisms and innocuous antigens (Ags) [21]. The oral mucosa is endowed with immune cells and soluble immune mediators that neutralize foreign Ags, limit colonization of pathogenic microorganisms, and mediate tolerance against commensal ones [22–24].

**Figure 2:**
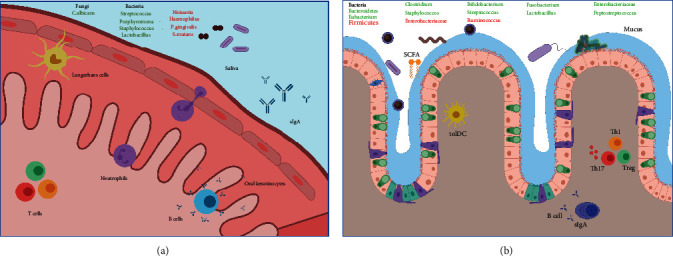
Oral and gastrointestinal microbiota. (a) The composition of oral microorganisms is implicated in maintaining homeostasis to keep the oral cavity in good health. Dysbiosis (red microorganisms) is associated with disease, but in addition, it can functionally contribute to the etiology, diagnosis, or treatment of the disease. (b) Metabolites derived from commensal bacteria interact with enterocytes and other intestinal cells. T cells localize in inguinal lymph nodes promoting class-switch and the production of secretory IgG by B cells. In dysbiosis and disease, pathogenic bacteria (in red) increase their abundance and induce a proinflammatory condition.

The expression of PRR, such as TLR o C-type lectin receptors on resident and innate immune cells, is pivotal for microbial, viral, fungal, and protozoal molecular pattern detection. This way, upon triggering of their signaling pathways, they mediate cytokine and chemokine secretion and express surface molecules for inducing an immune inflammatory or tolerogenic response [25]. PRR activation along with local microenvironmental mediators initiates and determines the specificity, sensibility, magnitude, and extent of the immune response. Innate immune cells, such as dendritic cells (DC), orchestrate Ag-specific immunity through PRR activation [26–28]. On human gingiva, four subsets of DCs have been identified: conventional DC types I and II (cDC1 and cDC2), plasmacytoid DCs (pDCs), and Langerhans cells (LCs). As professional antigen-presenting cells, DCs patrol the tissue and upon encountering antigen, migrate to draining lymph nodes to then present to CD4+ and CD8+ T cells [29]. Particularly, oral LCs correspond to local DCs residing at basal and suprabasal layers of the oral epithelium, offering permanent surveillance. In physiological conditions, TLR signaling induced by commensal microorganisms plays a key role in the maintenance of immune tolerance, epithelial homeostasis, and tissue repair (Figure 2). Peripheral immune tolerance is the lack of lymphocyte activation in response to Ags (anergy) or is the suppression of effector activity through the function of T regulatory cells (Tregs) [30–32].

Oral LCs have the capacity to produce the anti-inflammatory cytokines IL-10 and TGF-*β*, which suppress the activity of effector T cells. However, when the oral mucosa is invaded by pathogens, they initiate the adaptive immune response by generating exacerbated production of proinflammatory cytokines, which mediate the destruction of microorganisms and tissues [22, 33]. While mature DCs are potent activators of T cell responses, immature DCs mediate immune responses of low or anergic levels through the action of Tregs. In this context, when there is no inflammation, oral LCs and immature DCs from oral mucosa migrate to draining lymph nodes at a low rate, where they present commensal microorganisms Ags to naïve T cells and induce IL-10-producing T cells, which suppress immunity [34, 35]. On the other hand, in periodontitis-affected patients, in response to the oral microbiota and local inflammation, frequencies of gingival LCs and pDCs have shown to be deregulated, showing a decrease of LCs and expansion of pDCs [29]. These results uphold the relevance of DCs on the orchestration of both physiological and disease-associated immunity.

Humoral immunity also plays a key role in crosstalk with the microbiota. In the oral cavity, immunoglobulin A (IgA) in its secreted form (sIgA) is the predominant form of Ig which acts to limit the colonization and invasion of epithelium by microorganisms [36, 37].

Class switching provoked by T helper-dependent mechanisms generates IgA with high affinity for pathogenic microorganisms and toxins, facilitating their neutralization. Nonetheless, T helper-independent mechanisms give rise to IgA with low affinity for commensal microorganisms, limiting their growth and maintaining their homeostasis [38]. In addition, the presence of sIgA on the surface of oral mucosa contributes to immune tolerance mediating adhesion and neutralization of foreign Ags, which limits microorganisms' colonization [22, 23]. An example of these mechanisms is the control of *Candida albicans*, a commensal fungus that normally colonizes epithelial surfaces of oral mucosa without causing harm to the host. Oral keratinocytes and DC can distinguish between the yeast colonizing form or potentially invasive hyphae form via PRR [39, 40] and generate specific responses depending on the recognized fungal form. In particular, the hyphae state induces cellular responses such as Th17 [41], which mediates the secretion of IL-17 and recruits neutrophils in order to eliminate infection [42].

On the other hand, impaired levels or deficiency of sIgA may elicit oral dysbiosis. Chang et al. studied salivary microbiota in IgA-deficient (IgA KO) and wild-type (WT) mice. Particularly, they found an association between oral bacteria and periodontitis. Their results showed a decreased frequency of health-associated *Streptococcus* and increased percentages of disease-associated *Aggregatibacter*, *Actinobacillus*, and *Prevotella* on IgA KO mice, accompanied by significantly higher levels of alveolar bone loss [43].

Furthermore, Igs may be associated with acute humoral responses. An example of an IgA-mediated immune response is against coronavirus disease 2019 (COVID-19). Recent studies have shown that IgA is the dominant early neutralizing antibody which contributes to the SARS-CoV-2-specific humoral response. This viral infection promotes peripheral expansion of IgA plasmablasts with mucosal homing potential shortly after the onset of symptomatic disease. In this study, the authors showed that serum IgA concentrations decreased one month after symptom onset, but neutralizing IgA remained detectable in saliva for a longer time [44]. Moreover, salivary anti-SARS-CoV-2 IgA has been proposed as a biomarker of mucosal immunity against COVID-19, work that is still under development [45, 46].

In sum, commensal and pathogenic microorganisms may generate different interactions with resident or infiltrating immune cells, generating different responses: immune tolerance or inflammation, respectively [47].

### 1.4. Lung Microbiota

The lung was considered for many years to be a sterile organ; however, the use of culture-independent techniques has contributed to the identification and characterization of microbial communities in the lung and has evidenced its complexity [48, 49]. Still, lung microbiota presents less bacterial biomass compared to the lower gastrointestinal tract but exhibits considerable diversity [50]. It is characterized by the presence of bacteria of the phylum *Firmicutes*, *Bacteriodetes*, *Proteobacteria*, *Fusobacteria*, and *Actinobacteria*, while the species of *Prevotella (Bacteroidetes)*, *Veillonella*, *Streptococcus (Firmicutes)*, *Pseudomonas*, and *Haemophilus (Proteobacteria)* are the most characteristic in healthy individuals [51–53].

Since lungs are overexposed to external stimuli, this microenvironment is characterized by an immune tolerance milieu primarily maintained by airway epithelial cells that act as a main barrier with their mucociliary clearance, secretion of antimicrobial peptides, and cytokines and growth factors that mediate leukocyte recruitment by PPR activation [54] . In addition, studies suggest that lung resident pDCs and cDCs can exert a suppressive function and might contribute to lung tolerance [55, 56]; however, recent studies have suggested that DCs exert a stimulatory function, stimulating different Th phenotypes [56–58]. Alveolar macrophages can also exert a suppressive function that contributes to maintaining lung tolerance by producing prostaglandins and TGF-*β* that suppress T cell activation [59] and induction of Treg differentiation by secreting retinoic acid and TGF-*β* [60]. Also, tissue-resident macrophages display an intrinsic ability to promote the generation of induced Tregs that contribute to this tolerance by similar pathways [61]. There is increasing evidence in murine and human studies suggesting that lung microbiota also contributes to this immune tolerant environment [62]. For instance, reports where allergic airway inflammation was induced by sensitization and challenge with ovalbumin in pathogen-free (PF) and specific pathogen-free (SPF) mice showed an elevation of the total number of infiltrating lymphocytes and eosinophils in the airways of allergic GF mice in comparison with control SPF mice, and this increase could be reversed by recolonization of GF mice with the complex commensal flora of SPF mice [63]. Similarly, using SPF and GF mice exposed intranasally to lipopolysaccharide (LPS) showed an earlier and greater inflammation in the lungs of GF mice than those of SPF mice. TLR4 showed higher expression in lung tissue of GF than SPF mice, and lung explant stimulation with different TLR agonists showed greater inflammation under almost all GF conditions [64]. An example of human studies that complement the relation of lung microbiota and immune tolerance was described by Segal et al. Subjects without a known pulmonary disease showed that the basal level of lower airway mucosal Th17 immune activation was associated with compositional characteristics of local lung bacteria, demonstrating that a lung microbiome that had higher bacterial load and composition derived from the upper respiratory tract was associated with elevated activation in comparison to a microbiome with a lower bacterial load and taxa harvested by saline lavage and bronchoscope [65].

In pathological conditions, the lung environment can change significantly, creating optimal growth conditions for other bacterial species that leads to changes in lung microbiota abundance and composition [62]. These changes have been widely associated with the progression of chronic lung diseases such as asthma, chronic obstructive pulmonary disease (COPD), and chronic suppurative lung disease (CSLD). Asthma is the most common chronic disease in children. It is characterized by abnormal airway mucosa, inflammation, and transient wheezing. Asthmatic infant patients present a dysbiotic microbiota characterized by the presence of pathogens such as *Haemophilus* and *Neisseria* spp., accompanied by a reduction of commensal bacteria such as *Prevotella* and *Veillonella* spp. [66]. Chronic obstructive pulmonary disease (COPD) is characterized by persistent airflow limitation that is usually progressive, caused mainly by an enhanced chronic inflammatory response in the airways and the lung [67]. COPD patients show an increase in *Proteobacteria* in their respiratory tract; also, *Haemophilus influenzae*, *Moraxella catarrhalis*, and *Streptococcus pneumoniae* are important pathogens present in COPD acute exacerbations [68, 69]. CSLD includes conditions characterized by progressive lung damage and chronic productive cough, such as cystic fibrosis [70], where the presence of pathogens such as *Pseudomonas aeruginosa* has been described and associated with poor survival [71]; also, *Staphylococcus aureus*, *Haemophilus influenzae*, and *Burkholderia cepacia* complex have been described as important pathogens [72].

**Figure 3:**
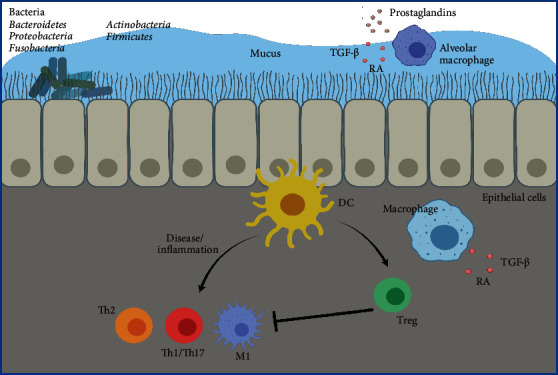
The immune tolerant microenvironment in the lung. Schematic representation of different components participating in lungs immune tolerance. Airway epithelial cells act as a primary barrier through mucociliary clearance and secretion of soluble factors. Alveolar macrophages have an immunosuppressive function, secreting prostaglandins and TGF-*β* and can also contribute to Treg differentiation by secreting retinoic acid and TGF-*β*. Lung microbiota such as Bacteroidetes, Proteobacteria, and Fusobacteria phyla also contribute to this tolerance by maintaining a healthy lung environment. Lung resident DC and macrophages keep an immunosuppressive milieu in healthy lung conditions; however, due to changes in lung environment, which may include variations in microbiota, inflammation status, or chronic lung diseases, an inflammatory state can be instead established.

In addition to lung dysbiosis, chronic lung diseases have also been associated with changes in the gut microbiome. For example, studies in patients with asthma have increased prevalence of irritable bowel syndrome (IBS) [73]. Additionally, studies have evidenced that low intestinal microbial diversity during the first month of life is correlated with the development of asthma during childhood [74]. These and other studies have suggested a gut-lung crosstalk in respiratory diseases, where gut dysbiosis (due to antibiotic use or diet composition) leads to an increased risk of developing pulmonary diseases or exacerbation of a preexisting one [75].  Figure 3 describes this section.

### 1.5. Gastrointestinal Microbiota

The gastrointestinal tract, with its epithelial barrier, presents a total area of 400 m^2^. It is open to and integrated with the most exposure to the external microenvironment. It contains at least 10^14^ microorganisms that belong to more than 2,000 species and 12 different phyla. Its microbiome contains 150 to 500 times more genes than the human genome [16].

Even though the interaction between the intestinal microbiota and the host cells is not fully understood, an important mechanism involves short-chain fatty acids (SCFA), like butyrate, acetate, and propionate, which correspond to bacterial products from undigested polysaccharide fermentation (dietary fibers). These SCFA have shown an important anti-inflammatory role in the maintenance of intestinal homeostasis (colon), participating in tissue repair through the promotion of cellular proliferation and differentiation (induction of Tregs and tolerogenic DC) [76].

The microbiota is also necessary for the immune system to mature and “learn” to differentiate between commensal and pathogenic bacteria [77]. In addition, TLR activation by intestinal microbiota Ags would result in inhibition of inflammatory reactions, which are essential for maintaining homeostasis [78]. It has been demonstrated that the intestinal microbiota modulates the migration and function of neutrophils [79] and would affect the differentiation of different T helper cells populations: Th1, Th2, and Th17 and Tregs [80]). Th17 cells are a subpopulation of CD4+ T cells that secrete multiple cytokines (such as IL-17A, IL-17F, and IL-22), with a significant impact on immune homeostasis and inflammation [81, 82]. It has been demonstrated that the administration of capsular polysaccharide isolated from the commensal bacteria *Bacteroides fragilis* suppresses IL-17 production and protects colonic mucosa from inflammatory reactions initiated by bacterial Ags, stimulating CD4+ T cells to produce IL-10 [83]. Interestingly, *B. fragilis* polysaccharide could stimulate TLR2 on Tregs for suppressing a Th17 response [84]. On the other hand, colon surroundings also stimulate the expansion of de novo generated Tregs derived from naïve CD4+ T cells [85].

Perturbations in the composition and function of bacterial and fungal intestinal microbiota have been associated with intestinal bowel diseases (IBD), including Crohn's disease (CD) and ulcerative colitis (UC) [86]. Both conditions exhibit a loss in intestinal bacterial diversity and expansion of specific bacterial families such as *Enterobacteriaceae* [87]. Moreover, the loss of certain symbiotic taxon like *Faecalibacterium prausnitzii* has been related to the appearance of CD, whereas the administration of these bacteria would reduce inflammation as shown in a chemically induced murine model of colitis, suggesting an anti-inflammatory role.

In the case of atypic asthma, there is evidence showing a relationship between environmental exposure, intestinal bacterial microbiota, and upper respiratory allergic pathology [88]. It was discovered that intestinal bacterial microbiota of mice treated with animal dandruff was enriched in *Lactobacillus johnsonii*, and oral supplementation with these bacteria protected animals against the induction of experimental allergy and respiratory infections, showing reduced concentrations of IL-4, IL-5, IL-13, and IL-17 in the upper respiratory airways, and a higher number of Tregs [89]. In another report, Maffeis et al. described that in children at risk of type 1 diabetes (T1D), increased intestinal permeability is correlated with microbiota alterations. In contrast to healthy controls, children at risk of T1D showed high levels of *Globicatella sanguinis*, *Dialister invisus*, and *Bifidobacterium longum* [90]. In addition, the *Bacteroidaceae* family was enriched in children with T1D, whereas a decrease in *Bifidobacterium pseudocatenulatum* and *Bifidobacterium adolescentis* was found [91].

It has been described that the intestinal microbiota has a role in the development of cancer. CD patients present a reduction in the abundance and a loss in the microbial equilibrium, which promotes an inflammatory state that increases the risk of neoplastic transformation. Moreover, various subproducts of the intestinal microbiota target intestinal epithelial cells, mediating oncogenic effects (as reported for hydrogen sulfur and *Bacteroides fragilis* toxin), enhancing the risk of colorectal cancer [92, 93]. Experimental alterations in the intestinal microbiota have demonstrated its influence in extra intestinal cancer's incidence and progression, including breast and hepatocellular carcinomas, presumably through inflammatory and metabolic mediators. These results are compatible with those described in epidemiological studies, which reveal an association between dysbiosis, its consequences, or causes (specially, the use of antibiotics), and a higher incidence of extracolonic neoplasia, including breast carcinoma. Figure 2 summarizes these findings.

Riquelme et al. reported that patients with different stages of pancreatic adenocarcinoma present variations in intratumoral microbiota diversity. Interestingly, this microbiota seems to communicate with the intestinal one, which could be influencing the host immune response and the disease [94–96].

### 1.6. Genitourinary Microbiota

**Figure 4:**
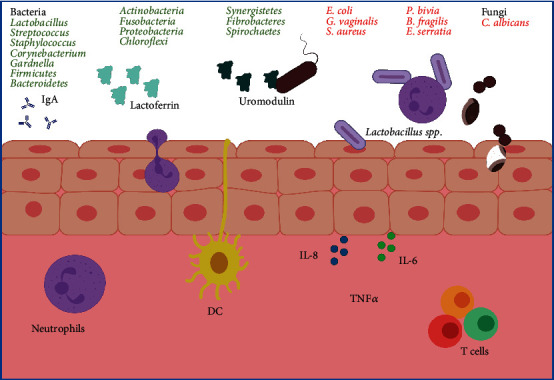
Main components present in the genitourinary microbiota. The main cells of the immune system that are present in the genitourinary mucosa are shown, in addition to relevant molecules such as IgA antibodies and key proteins of a healthy mucosa. Commensal microorganisms of the genitourinary tract are indicated in green and pathogenic microorganisms in red.

The healthy maintenance of the genitourinary system in women is essential to lead a good quality of life, both sexual and reproductive. A healthy vaginal environment requires an optimal interaction between the individual and the vaginal microbiota, which is mainly composed of *Lactobacillus* spp. [97, 98]. A study describes the complexity of the vaginal microbiota using massive sequencing technology, where 400 women of different ethnicities of childbearing age were analyzed, identifying a predominance of the following lactobacillus species: *Lactobacillus crispatus*, *Lactobacillus jensenii*, *Lactobacillus iners*, and *Lactobacillus gasseri*. In addition to lactobacillus, bacteria such as *Streptococcus*, *Staphylococcus*, *Corynebacterium*, and *Gardnerella* can be found, as well as others from the intestine, which in low amounts do not disrupt homeostasis (Figure 4). The vaginal microbiota evolves and changes throughout a woman's life with age and hormonal status, with significant alterations during the menstrual period, pregnancy, and the puerperium or menopause [99].

The vaginal microbiota adheres specifically to the vaginal walls and the cervix, forming a biofilm that does not allow the adhesion of unwanted microorganisms, contributing to the integrity of the mucous. It also competes with other microorganisms that could potentially be pathogens, preventing their attachment to the mucous membranes so that they cannot infect or reducing their nutritional substrate. Importantly, this biofilm degrades foreign substances that can be harmful to the environment. Furthermore, they produce antimicrobial substances and aggregates with pathogens, forming structures that facilitate the effect of microbial substances that they release. One of the antimicrobial molecules corresponds to lactic acid, which reduces the pH of the medium and prevents the growth of pathogens, especially those that come from the intestine. Even more, the production of hydrogen peroxide stops the growth of germs such as gonococcus, a typical sexually transmitted infection. The most common causes for the decrease in lactobacilli are the abuse of antibiotics, stress, smoking, excessive hygiene with vaginal douches, etc. In this regard, vaginal probiotics have been developed to restore the loss of lactobacilli and improve vaginal immunity [99, 100].

The most common pathology in women is bacterial vaginosis (BV), which is described as a polybacterial dysbiosis, where *Lactobacillus* load decreases, and both the diversity and the bacterial load of other anaerobic (facultative) bacteria increase. Vaginal dysbiosis is a very common condition that affects immune homeostasis, induces a breakdown in the epithelial barrier, and favors infection by sexually transmitted pathogens. The microorganisms most associated with this dysbiosis are *Gardnerella vaginalis*, *Atopobium spp.*, *Mobiluncus spp.*, *Prevotella bivia*, *Bacterioides fragilis*, *Candida albicans*, *Escherichia coli*, and *Staphylococcus aureus* [100].

In vitro studies have shown that certain *Lactobacillus* species can attenuate inflammation by reducing the secretion of IL-6, IL-8, and TNF-*α* after bacterial stimulation of TLR; in fact, it is assumed that a poor ecosystem in *Lactobacillus* leads to a greater probability of contracting sexually transmitted infections.

Under normal conditions, the urinary tract microbiota is made up of 20 to 500 bacterial species distributed in nine major phyla: *Firmicutes, Bacteroidetes*, *Actinobacteria*, *Fusobacteria*, *Proteobacteria* and to a lesser extent *Chloroflexi*, *Spirochaetes*, *Synergistetes*, and *Fibrobacteres*. Urinary tract infections are mainly caused by pathogens of intestinal origin that contaminate the urethra and ascend to the bladder; however, a variety of bacteria, fungi, yeasts, viruses, and parasites can also cause urinary tract infections. About 90% of urinary infections are Gram (-) bacilli of the *Enterobacteriaceae* family, reaching this area from the urethra colonized by the fecal flora of the digestive tract. *Escherichia coli* is the most frequently implicated and mainly responsible for pyelonephritis and cystitis. Other opportunistic microorganisms such as *Proteus*, *Serratia*, or *Pseudomonas*, and fungi, especially *Candida albicans*, whose pathogenic action is favored by the presence of debilitating diseases, immunosuppression, and surgical interventions, also cause infections [97, 99].

In urinary tract infections by *E. coli*, the cell-bacterial interaction would stimulate a process of apoptosis and the shedding of protective cells from the epithelium, which remains exposed to a new cycle of infection, being able to remain in a quiescent state for months. Lactoferrin, uromodulin, IgA antibodies, cathelicidin, and defensins are secreted by various host cell types to inhibit the adhesion of E. coli to the epithelium. The production of IL-6 and IL-8 stimulates the migration of immune cells, mainly neutrophils. Activation of Ag-presenting cells allows activation of T cells and differentiation of B cells to plasma cells that produce immunoglobulins. IgA inhibits bacterial adherence and neutralizes enzymes, viruses, and toxins [97, 100].

One of the most studied stages of the uterine microbiota is pregnancy and its direct relationship with the immune system because it is believed that pregnancy protects against invading pathogens as well as tolerance of and support of implantation and growth of the semiallogeneic fetus. It has been reported that the first phase of a zygote's implantation is characterized by presenting a low-grade proinflammatory reactivity, releasing mainly IL-6, IL-8, and TNF-*α* [101].

On the other hand, the first line of defense of the female reproductive system against pathogens is the physical barrier mainly composed by a layer of mucous, IgA antibodies, and commensal bacteria that help limit the colonization of pathogenic bacteria [101]. In addition, it has been reported that NK cells play an important role in the female reproductive system by protecting the vagina from a wide variety of viruses [102].

On the other hand, the microbiota of the male genitourinary system is scarce because urine washes the urethra periodically and because the exit orifice is widely separated from the anus, which is the main source of contamination of the excretory system. Some recent research has questioned the bladder being a sterile environment, and that urinary tract infections are related to bacteria of intestinal origin. It is currently believed that these infections could be caused by dysbiosis of the urinary microbiota, especially in certain pathologies of the urinary tract or of the prostate. The urinary tract does not have an autochthonous microbial flora, except for the distal portion of the urethra, which can be colonized by the normal microbiota of the skin. In the urine of healthy individuals, we can find saprophytic microorganisms or those carried in the urine such as *Lactobacillus*, *Bacillus*, *Corynebacterium*, *Staphylococcus*, *Candida*, and some *Enterobacteriaceae* [97].

### 1.7. Skin Microbiota

The skin, with an estimated surface of 2 m^2^, is considered the biggest organ of the body, being involved in diverse functions such as immune protection, hydrosaline equilibrium, thermoregulation, and metabolism, among others. Lately, the role of microbiota has been investigated in several pathologies, which has been facilitated by modern 16S ribosomal sequencing techniques, permitting the finding of undetectable species that cannot grow in traditional cell cultures [103].

The skin microbiota generated at the time of birth is vital for the generation of tolerance to species that will later populate this organ. This process is mediated mainly by Tregs [86]. This way, a wide variety of microorganisms is established, and its proportion varies with age as reported by Oh et al. when comparing the bacterial flora in subjects aged from 2 to 40 years [104]. Skin's conditions, such as pH, humidity, temperature, and thickness, determine the species present in it. For instance, humid places like armpits or interphalangeal folds of the foot predominate *Staphylococcus* spp. and *Corynebacterium* spp.; other places such as those rich in sebaceous glands *Propionibacterium* spp. and *Malassezia* spp. are mainly found, and in dry zones as arms or legs, the variety includes *Propionibacterium* spp., *Corynebacterium spp.*, *Streptococcus spp.*, and even Gram (-) bacteria [105]. However, this prevalence seems to change among healthy individuals due to the influence of the environment and the individual's genetic make-up, adding an interpersonal variation to the nature of the skin microbiota [16, 106].

Lately, the importance of the skin microbiota in the immune system's activation and disease has been highlighted. For example, the release of *δ*-toxin by *S. epidermidis* controls the proliferation of pathogens such as group A *Streptococcus* (86). Others, like nisin or epidermin lantibiotics, are produced by commensal and pathogenic bacteria (like *S. aureus*) to compete for their place [107]; in addition to phenol-soluble modulins, a class of potent cytolysin traditionally associated with *S. aureus*, whose production has also been reported in *S. epidermidis* [108]. This could be relevant in the context of atopic dermatitis flares, where the representation of commensal *S. epidermidis* is significantly increased, as described by Kong et al., performing 16S ribosomal RNA bacterial gene sequencing from serial skin sampling of human patients [109].

**Figure 5:**
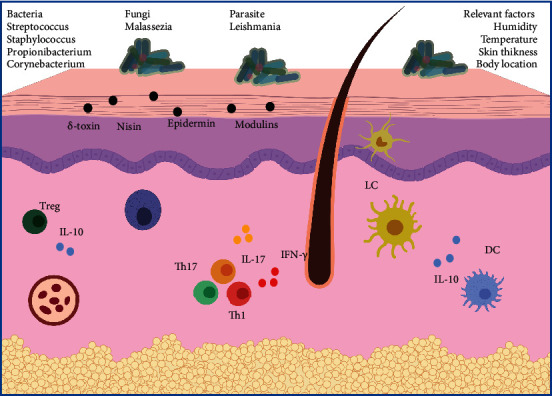
Microbiota, cells, molecules, and environmental factors in skin status. Through the release of mediators, such as modulins, and the participation of immune cells and molecules, the skin controls its own homeostasis. Environmental factors like pH, humidity, temperature, skin thickness, and body location actively affect the composition of this organ.

The microbiota not only interacts with pathogenic microorganisms to exert an immunological function but with the host cells as well. For instance, animal studies have shown that the presence of *S. epidermidis* activates IL-17 and IFN-*γ* secreting T cells through IL-1 and MyD88-dependent mechanisms, whereas its absence downregulates the activation of these pathways resulting in higher numbers of Tregs. This scenario is found during infection where the absence of microbiota is associated with a poor response against infections caused by *Leishmania major* [110]. From the above, we can infer the relevance of commensal microbiota in the activation of effector responses against local pathogens. However, this interaction could also have a role in the control of inflammation. In this regard, it has been observed that *S. epidermidis* suppresses the immune response after skin injury through lipoteichoic acid release, which binds to TLR2 on keratinocytes, diminishing the release of proinflammatory cytokines that occurs via TRAF1 [111]. Similarly, it has been observed that environmental microbiota can also influence the immune system. For instance, *Vitreoscilla filiformis*, a Gram (-) bacteria found in thermal waters, has been used to control inflammation in atopic dermatitis patients through the stimulation of DC-derived IL-10 release, which in turn activates Tregs [112]. In the case of commensal bacteria that turns into pathogenic agents, studies involving *C. acnes* indicate that healthy individual host strains trigger the release of anti-inflammatory cytokines such as IL-10, whereas patients have proinflammatory strains that stimulate the production of cytokines like IL-17 and IFN-*γ* [113]. In the case of seborrheic dermatitis, there is a close relationship between an improvement in the condition and a reduction in levels of *Malassezia* spp. in the scalp, as seen when comparing the effects of various antifungals [114]. An overview of the above is presented in Figure 5.

Finally, a crosstalk between intestinal microbiota and skin pathologies has been suggested. Studies in animals reported that administration of probiotics, which augments microbial intestinal biodiversity, induces a tolerogenic microenvironment determined by an increase in Tregs, which finally translates into clinical benefits in diseases such as atopic dermatitis [115]. However, studies in humans with atopic dermatitis show that despite an elevation of the fecal cell count after the use of probiotics, there is no evidence of significant clinical improvement or changes in levels of serum cytokines such as TNF-*α*, IL-4, or IL -10 compared to placebo [116]. Despite the above, studies of serial human fecal samples using 16S sequence techniques reflect significant differences in microbial diversity between healthy children and those with eczema at early ages, with less bacterial variability in the latter [117].

## 2. Conclusions

Current information shows that the relationship between microbiota and the immune system is essential for the maintenance of a healthy state and for resolving pathological situations. Future technological advances and research in this area will contribute to comprehending how these anatomical sites are regulated; thus, novel interventions can be applied in disease.

## Data Availability

No data were used to support this study.
